# Supplementary material for: Control of RAB7 activity and localization through the retromer‐TBC1D5 complex enables RAB7‐dependent mitophagy
Source: EMBO J. 2017 Nov 20;37(2):235–54. doi: 10.15252/embj.201797128 (PMC5770787; doi:10.15252/embj.201797128)
Supplement: Supplementary file 1 — Expanded View Figures PDF [file EMBJ-37-235-s001.pdf]

Expanded View Figures

Figure EV1. RAB7 localizes to mitochondria and the ER.

All images show formaldehyde-fixed HeLa cells.

A HeLa cells stained for endogenous RAB7a (green) and endogenous LAMP2 (red). White arrows indicate sites of vesicular RAB7 that appears to be budding from a network of RAB7.
B Immunofluorescent co-staining of endogenous RAB7a (green) and the mitochondria marker TOM20 (red).
C Co-staining of endogenous RAB7 (green) and the *trans*-Golgi network marker TGN46 (red).
D Co-staining of endogenous RAB7 (green) and the ER marker protein disulfide isomerase (PDI, red).
E Co-staining of lentivirally expressed GFP-RAB7a and endogenous TOM20 (red).
F Co-staining of endogenous RAB7 (green) and TOM20 (red) in cells treated with Cas9 and a mixture of three gRNAs targeting the RAB7a locus. The Western blot of this cell population shows an almost complete loss of RAB7 in the treated cells compared to parental HeLa cells.
G GFP-RAB7 was expressed in RAB7a KO HeLa cells and imaged in live cells using a spinning disk confocal microscope. Mitochondria were visualized with MitoTracker Red.
H Parental HeLa cells and clonal VPS29 KO cells were co-stained for endogenous RAB7a (green) and endogenous TOM20 (red), and co-localization was analyzed across two independent experiments.

Data information: All scale bars = 10 μm, all error bars = SD, and $^*P < 0.05$ in a t-test of the respective condition compared to the control cells.
Source data are available online for this figure.

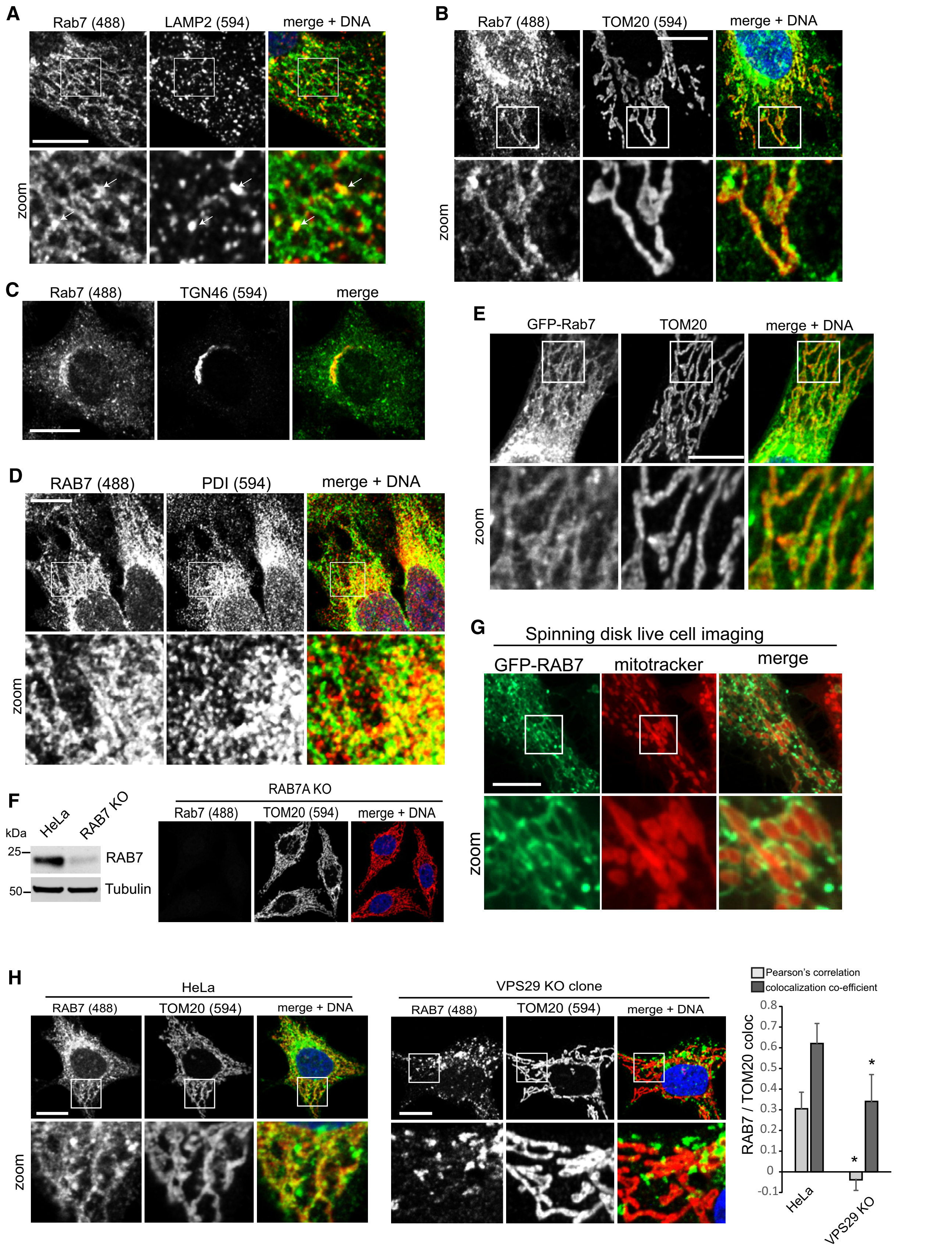

Figure EV1.

 © 2017 The Authors

Figure EV2. RAB7 activity state controls its localization to the ER, mitochondria, and lysosomes.

A Mitochondria were purified from detergent-free postnuclear supernatants using magnetic beads coated with a TOM22-specific antibody from parental HeLa cells and from clonal VPS35 KO cells. The purified mitochondria were then subjected to a marker analysis using the indicated organelle markers. Note that only mitochondria (TOM20) and RAB7 are enriched over the inputs, with lower levels of RAB7 precipitating with the mitochondria of VPS35 KO cells. The quantification of RAB7 was done across three independent mitochondria isolations.

B RAB7a KO cells transduced with the indicated GFP-RAB7a variants were fixed and co-stained for endogenous LAMP2. Note that the inactive RAB7 (T22N) does not localize to lysosomes, whereas the hyperactive variant (Q67L) fully localizes to lysosomes.

C, D RAB7a KO cells transduced with the indicated GFP-RAB7a variants and a mCherry-ER marker were fixed and co-stained for endogenous TOM20.

E RAB7a KO cells were infected with the indicated GFP-RAB7 variants, labeled with MitoTracker Red, and subjected to live cell imaging using a spinning disk confocal microscope.

Data information: All scale bars = 10 μm, and all error bars = SD.
Source data are available online for this figure.

© 2017 The Authors

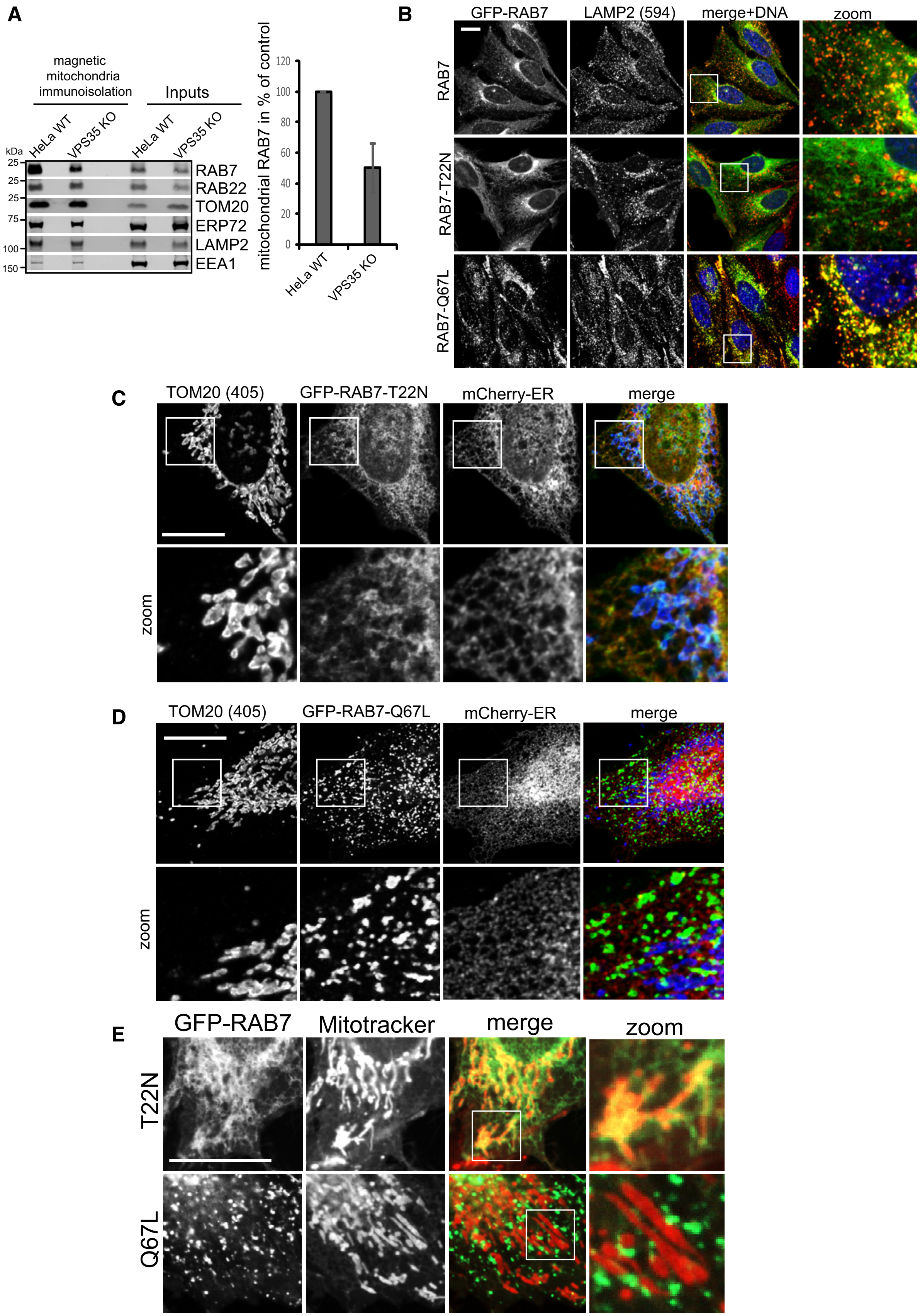

Figure EV2.

 © 2017 The Authors

Figure EV3. Retromer controls RAB7 activity levels.

A, B Parental HeLa cells and clonal VPS35 KO cells were infected with lentiviruses expressing inactive GFP-RAB7-T22N (A) or constitutively active GFP-RAB7-Q67L (B) and co-stained for endogenous TOM20 (red). Co-localization was analyzed across two independent experiments.

C RILP effector assay comparing VPS35 KO cells and VPS35 KO cells that had been infected with a lentivirus encoding untagged human VPS35.

D Immunofluorescent staining showing that lentivirally expressed VPS29-myc restores vesicular VPS29.

E RILP effector assay with lysates from parental HeLa cells and VPS35 KO cells and lysates from VPS35 KO cells that were spiked with recombinant VPS35 produced in bacteria. Note that re-addition of VPS35 has no effect on the amount of active RAB7 binding to the GST-RILP beads.

F Quantitative proteomic interactome analysis of GFP-RAB7 isolated from parental and VPS35 KO cell lines. The SILAC ratios (from two independent experiments with swapped isotope labeling) for the indicated proteins suggest that RAB7 is more active in the knockout cells, as the RAB7 effector RILP is increased in the RAB7 IP from KO cells while the RAB chaperones GDI1, GDI2, and PRA1 are strongly decreased.

G HeLa cells were fixed in cold methanol and stained for endogenous RAB7a and LAMP2. The same cell and same *Z*-position was then imaged at low-laser settings (left) and at high-laser setting on a confocal microscope.

Data information: All scale bars = 10 μm, and all error bars = SD.
Source data are available online for this figure.

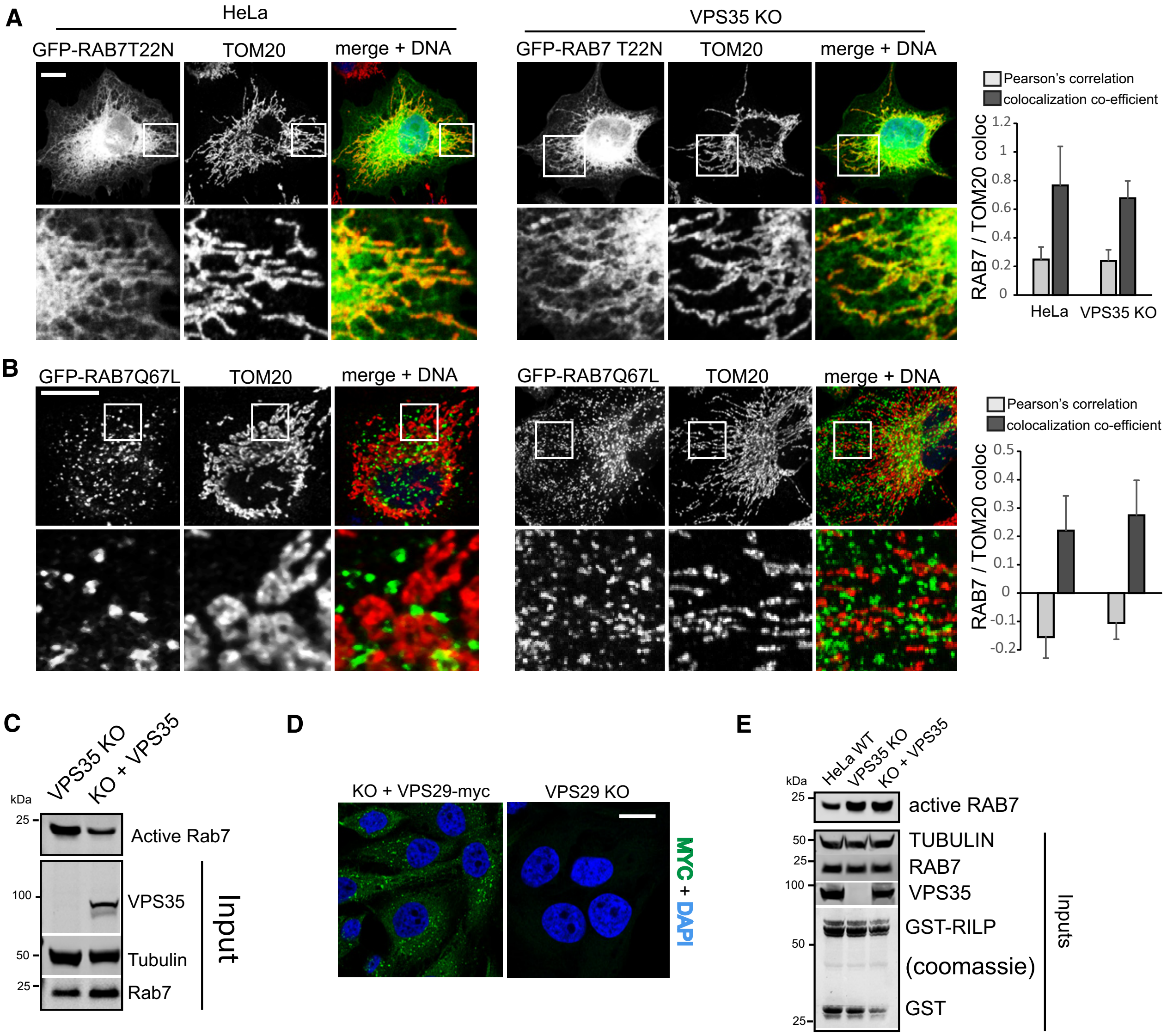

F

Protein	fold-enrichment WT/VPS35 KO	fold-enrichment VPS35KO/WT
Rab-interacting lysosomal protein	0.51	3.89
Rab GDP dissociation inhibitor alpha	6.3	0.16
Rab GDP dissociation inhibitor beta	13.5	0.08
Prenylated Rab acceptor protein 1	6.30	0.16263

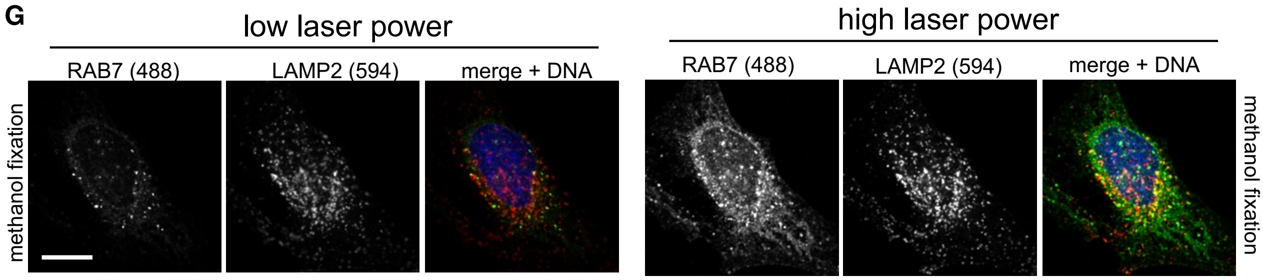

Figure EV3.

 © 2017 The Authors

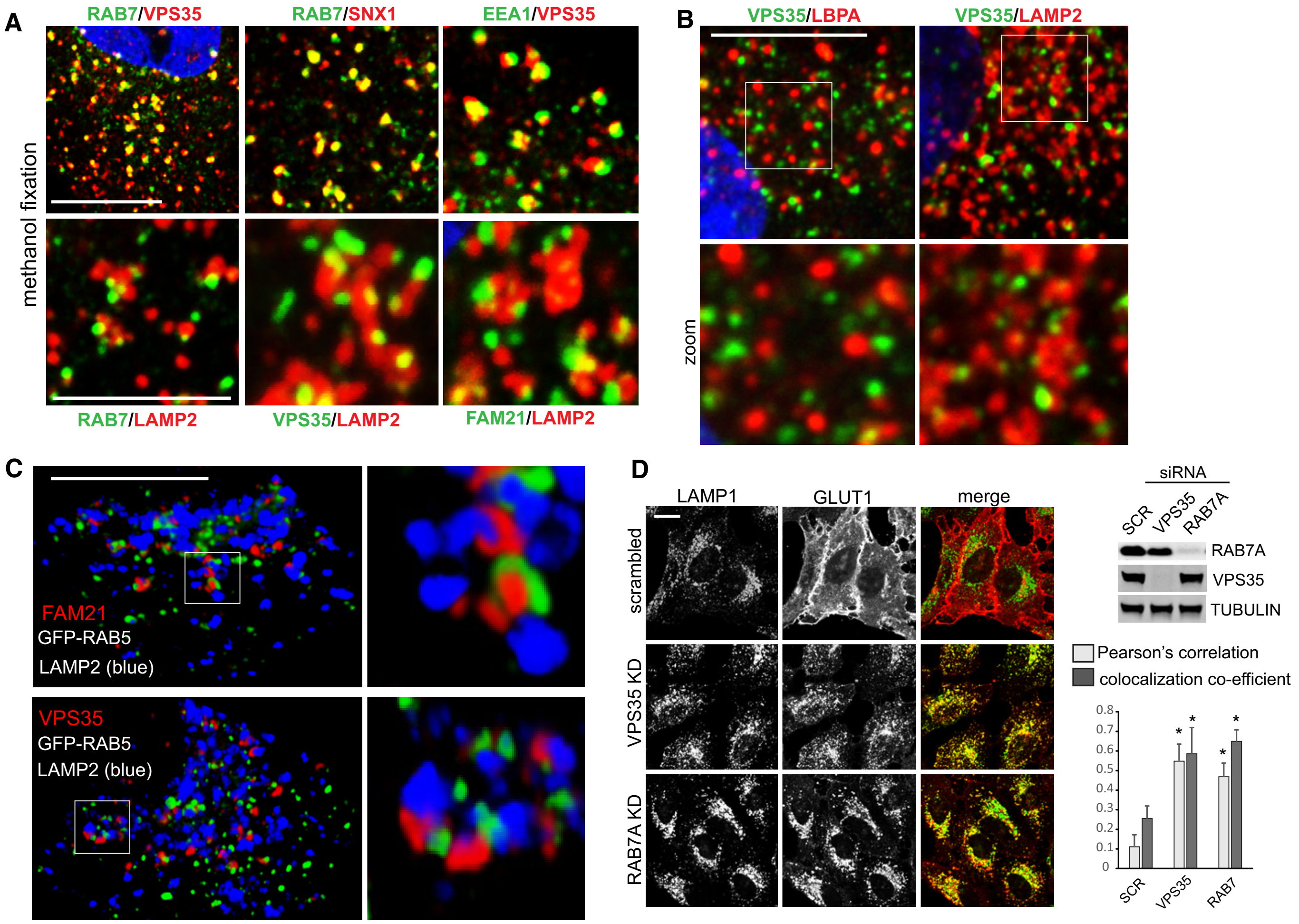

Figure EV4. RAB7 and retromer are a functional unit at the interface between endosomes and lysosomes.

A HeLa cells fixed in methanol were co-stained for the indicated endogenous proteins. Green signal is Alexa 488, and red signal is Alexa 594.

B Co-staining of endogenous VPS35 and the late endosome marker LBPA (left) and the late endosome/lysosome marker LAMP2. Note that VPS35 seems to be less associated with LBPA-positive domains than with LAMP2-decorated entities.

C 3D reconstruction of PFA-fixed single cells expressing GFP-RAB5 and co-stained for endogenous LAMP2 (Alexa 405, blue) and endogenous VPS35 or FAM21 (Alexa 594, red).

D HeLa cells transfected with siRNA targeting VPS35 and RAB7a were co-stained for the endogenous glucose transporter GLUT1 (Alexa 594, red) and the lysosomal marker LAMP1 (Alexa 488, green). Efficiency of the siRNA was tested by Western blotting. Co-localization was analyzed over two independent experiments with 10 images each.

Data information: All scale bars = 10 μm besides lower panel in (A), where scale bar = 5 μm, all error bars = SD, and *$P < 0.05$ in a t-test of the respective condition compared to the control cells.
Source data are available online for this figure.

Figure EV5. Loss of TBC1D5 does not perturb retromer-mediated sorting of GLUT1 and CI-MPR.

A HeLa, SNX5/6 double KO, and TBC1D5 KO cells were fixed in PFA and stained for CI-MPR (red) and the *trans*-Golgi network marker TGN46. Note that knockout of SNX5/6 results in dispersal of CI-MPR from the TGN.

B HeLa, VPS29, and TBC1D5 KO cells were fixed in PFA and stained for GLUT1 (green) and LAMP2 (red). Co-localization between GLUT1 and LAMP2 was quantified over ten images for each condition.

C HeLa cells were transfected with a pool of three distinct CRISPR/Cas9 plasmids targeting the RAB7a gene at three sites together with a puromycin resistance plasmid. Following puromycin selection and 5 days of incubation, the RAB7a KO cells were transduced with lentiviruses expressing the indicated GFP-RAB7 proteins. A Western blot for RAB7 confirms high knockout efficiency and demonstrates that the GFP-RAB7 variants are expressed at low endogenous levels.

D Parental HeLa cells and clonal VPS29 and VPS35 KO cells transduced with GFP-LC3b were starved in EBSS for 4 h without (EBSS) or with addition of bafilomycin A1 to the EBSS (EBSS + Bafi) to assess autophagic flux. Autophagic flux was calculated from the increase in lipidated GFP-LC3 in the EBSS + bafilomycin samples compared to the EBSS-only sample. The quantification was done across four independent experiments.

Data information: All scale bars = 10 μm, all error bars = SD, and *$P < 0.05$ in a t-test of the respective condition compared to the control cells.
Source data are available online for this figure.

▶

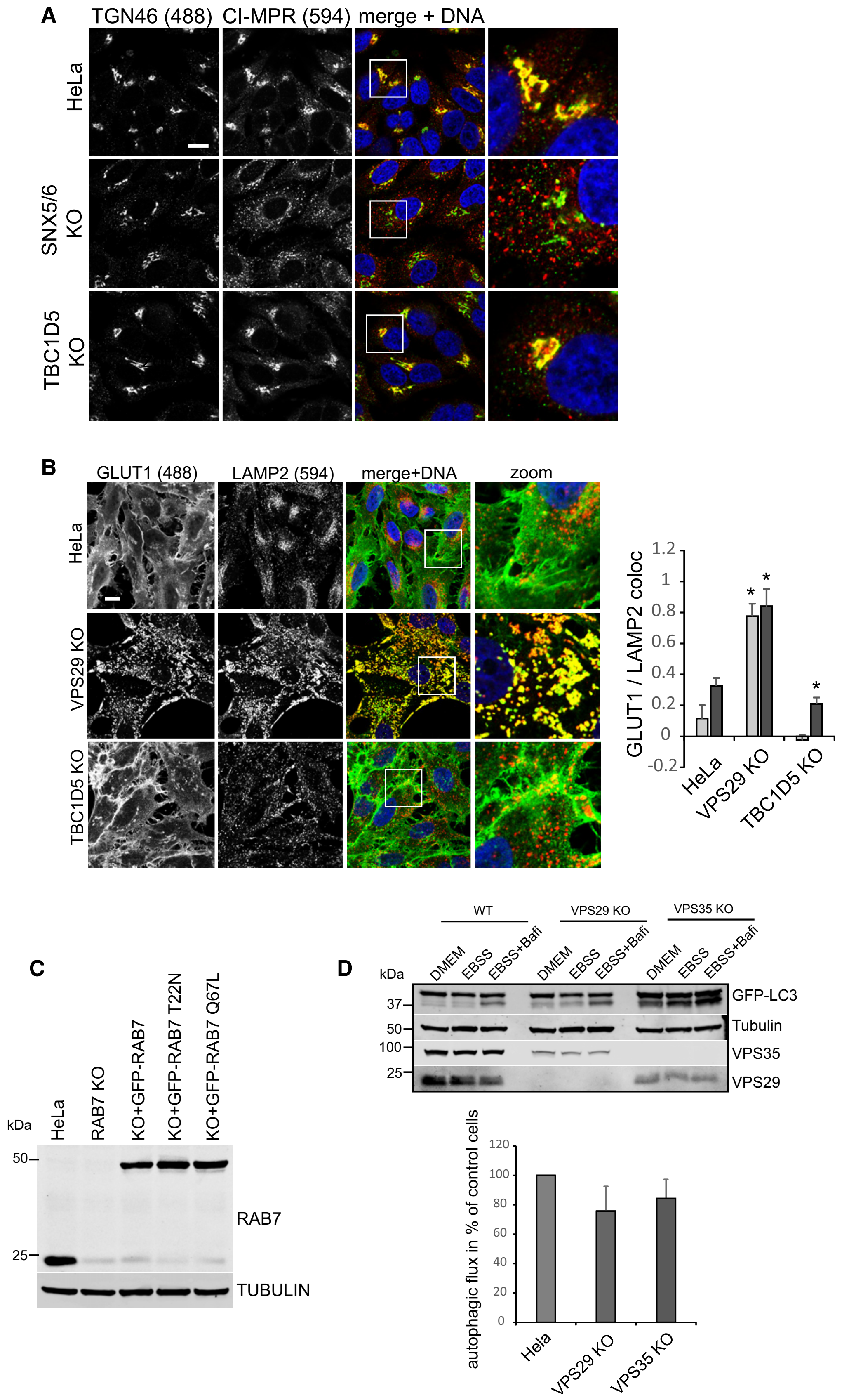

Figure EV5.

 © 2017 The Authors

Figure EV6. TBC1D5 mitophagy assay and assessment of the mCherry-GFP-FIS1TM mitophagy sensor.

A Parental HeLa cells and TBC1D5 KO cells transduced with the indicated GFP-TBC1D5 lentiviruses were infected with mCherry-Parkin, and mitophagy was induced overnight with the proton uncoupler CCCP. Clearance of mitochondria was assessed by staining for endogenous TOM20. Note that GFP-TBC1D5 rescued the TOM clearance defect in the TBC1D5 KOs, whereas the retromer-binding-deficient GFP-TBC1D5-L142E did not. The amount of residual TOM20 was assessed as the sum of the signal in the 405 channel in Volocity. The graph shows the average of ten images per condition.

B The mitophagy sensor mCherry-GFP-FIS1TM was transduced into HeLa cells together with HA-tagged Parkin. Mitophagy was then induced by treatment of the cells with CCCP for 6 h and for 16 h. The cells were then analyzed for red-shifting of the mCherry-GFP tandem label due to lysosomal quenching of the GFP moiety. Note that CCCP induced pronounced red-shifting, whereas no such shifting is detectable without CCCP, indicating that the sensor does indeed detect mitophagy.

Data information: All scale bars = 10 μm, and all error bars = SD.

© 2017 The Authors

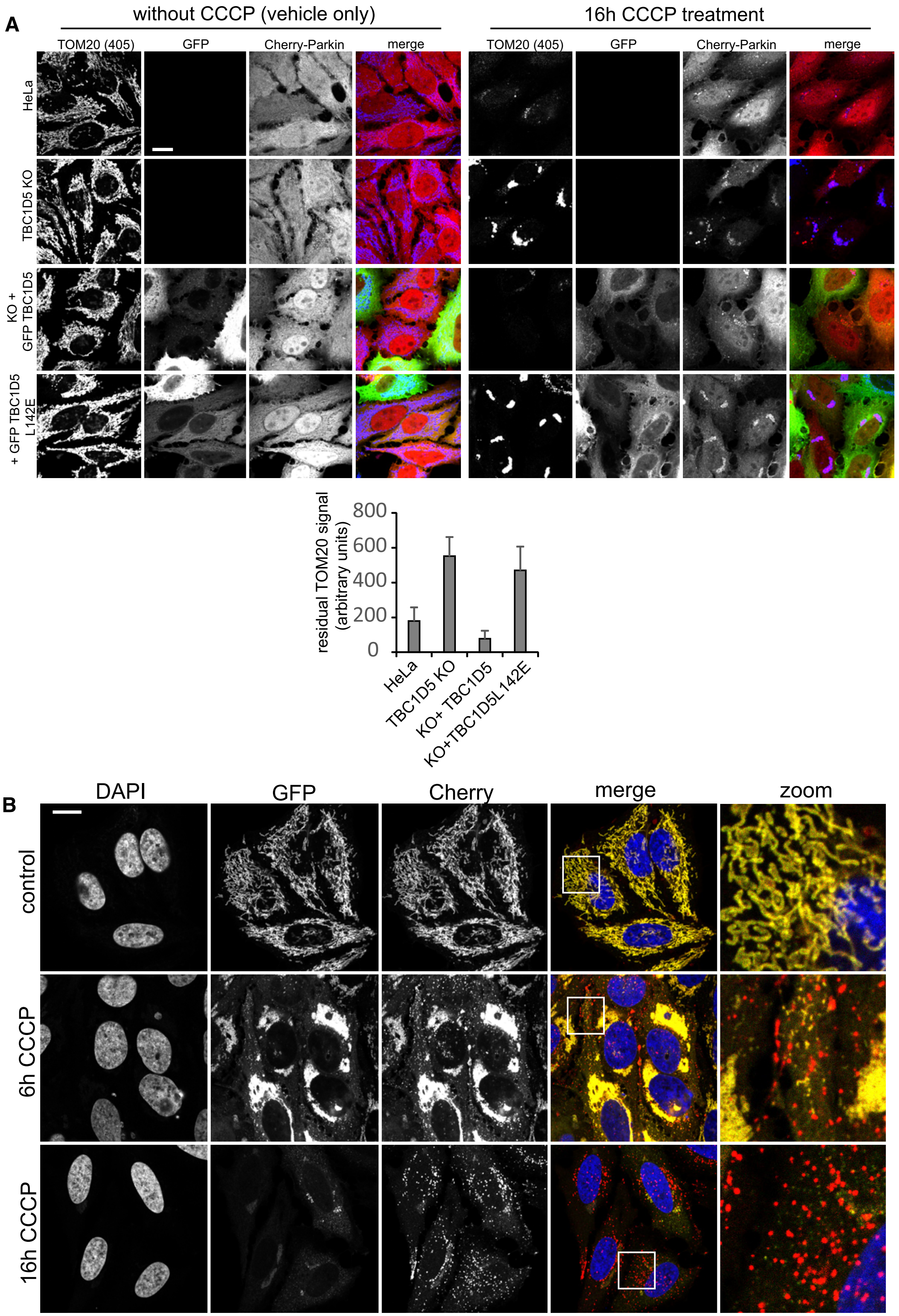

Figure EV6.

 © 2017 The Authors

Figure EV7. Additional mitophagy data.

A Parental HeLa cells and VPS29 and VPS35 KO cells transduced with mCherry-Parkin were treated with CCCP for the indicated time points, followed by staining of endogenous ATG16L1 (upper row) and endogenous TOM20 (lower row).

B Parental HeLa cells and VPS29 and VPS35 KO cells transduced with mCherry-Parkin were treated with CCCP for the indicated time points, followed by staining of endogenous ULK1 (lower row) and endogenous TOM20 (upper row).

C mCherry-Parkin-expressing parental HeLa cells, VPS35 KO cells, and RAB7a KO cells transduced with RAB7-Q67L were fixed and stained for endogenous ATG9a (green) and TGN46 (blue).

D Parental HeLa cells and VPS35 KO cells were transduced with mCherry-Parkin and GFP-RAB7, followed by fixation and staining of endogenous ATG9a (blue). Co-localization of ATG9a and GFP-RAB7 was analyzed across 32 individual cells acquired in two independent experiments.

Data information: All scale bars = 10 μm, all error bars = SD, and $^*P < 0.05$ in a *t*-test of the respective condition compared to the control cells.

© 2017 The Authors

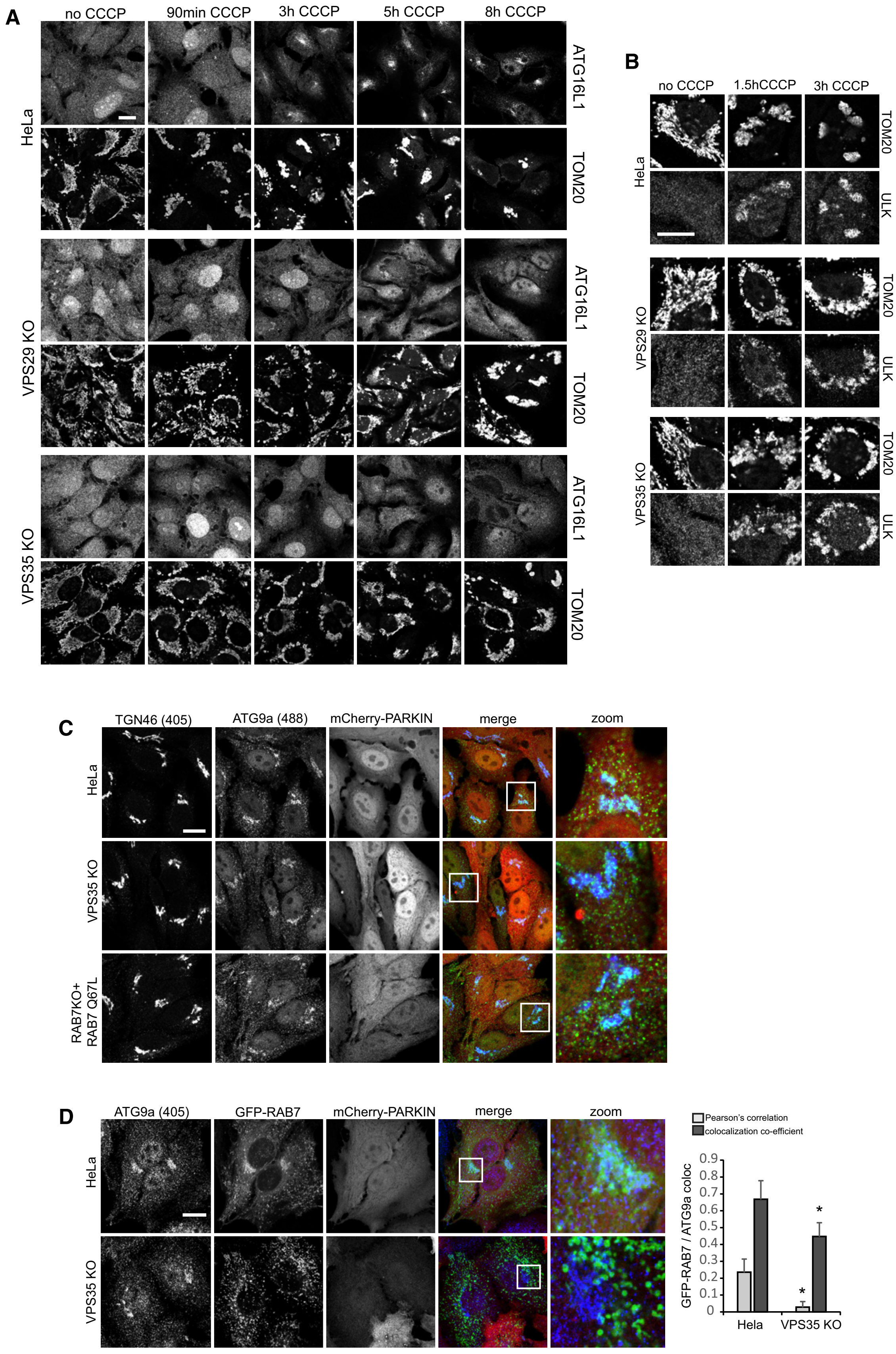

Figure EV7.

 © 2017 The Authors
