# Supplementary material for: Control of RAB7 activity and localization through the retromer‐TBC1D5 complex enables RAB7‐dependent mitophagy
Source: EMBO J. 2017 Nov 20;37(2):235–54. doi: 10.15252/embj.201797128 (PMC5770787; doi:10.15252/embj.201797128)
Supplement: Supplementary file 5 — Source Data for Expanded View [file EMBJ-37-235-s011.zip › Figure_EV3_blot_data.pdf]

Figure EV3C: VPS35 rescue RILP

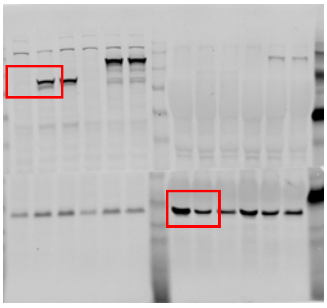

VPS35 and RAB7 beads

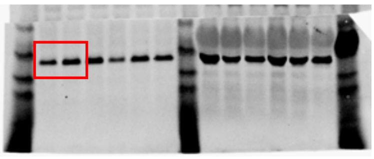

RAB7 input (higher expo)

same membrane as VPS35 blot above,
Tubulin in 680nm channel

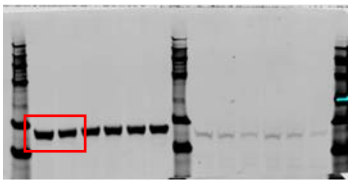

Tubulin

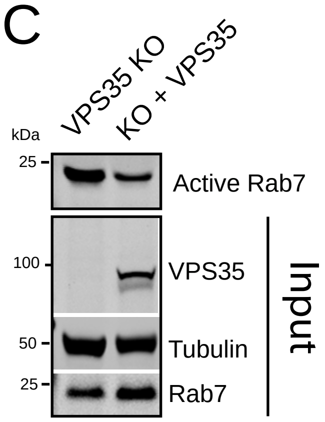

Figure EV3E: recombinant VPS35 in RILP assay

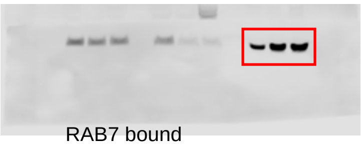

RAB7 bound

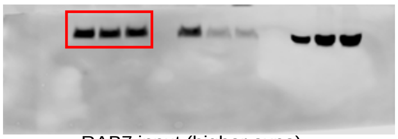

RAB7 input (higher expo)

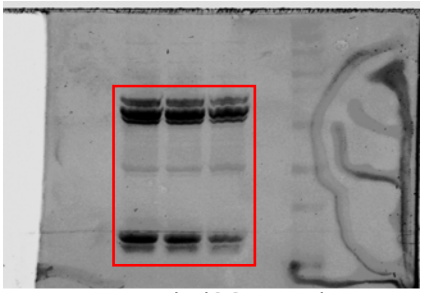

coomassie (GST-RILP)

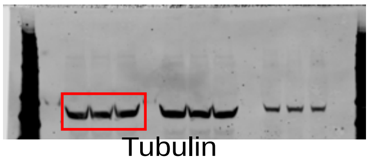

Tubulin

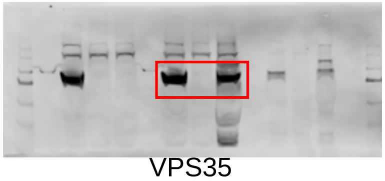

VPS35

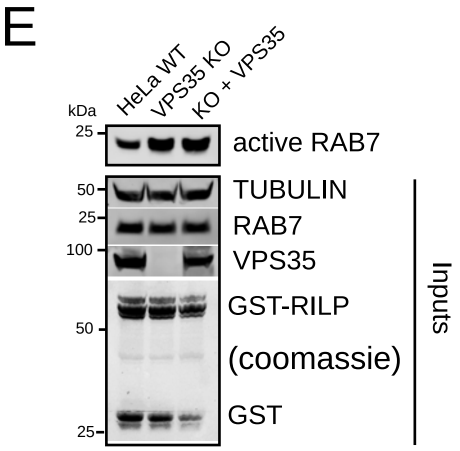

For VPS35, Tubulin and RAB7 blots: Note that the three lanes on the left are the input lysates before addition of recombinant VPS35, whereas the three middle lanes are from the flow through after VPS35 was added and RAB7 was pulled out with the GST-RILP beads.
