# Supplementary material for: Control of RAB7 activity and localization through the retromer‐TBC1D5 complex enables RAB7‐dependent mitophagy
Source: EMBO J. 2017 Nov 20;37(2):235–54. doi: 10.15252/embj.201797128 (PMC5770787; doi:10.15252/embj.201797128)

Figure EV4A: RAB7a KD control

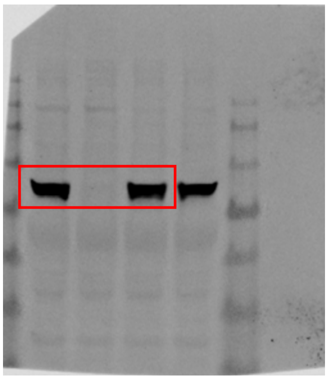

VPS35

same membrane as above with
Tubulin in the 680nm channel:

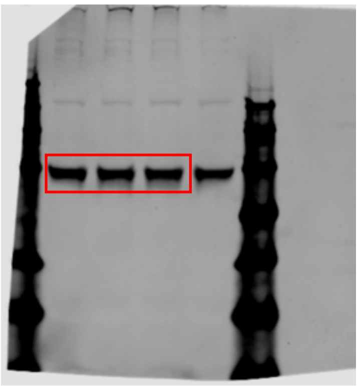

Tubulin

membrane fragment cut off from membrane above
and incubated with RAB7 antibody

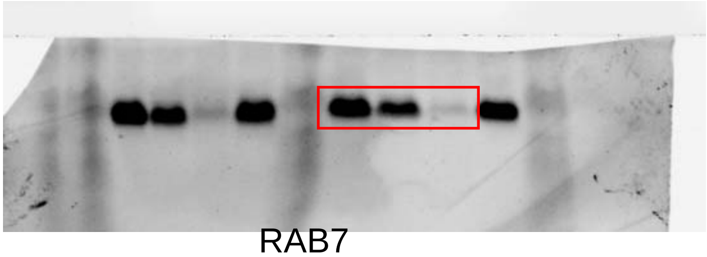

RAB7

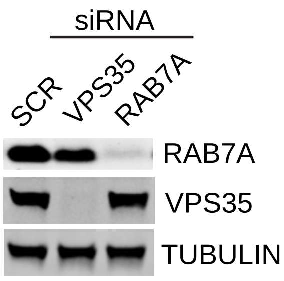

Supplement: Supplementary file 5 — Source Data for Expanded View [file EMBJ-37-235-s011.zip › Figure_EV4__blot_data.pdf]
