# Supplementary figures and images for: Control of RAB7 activity and localization through the retromer‐TBC1D5 complex enables RAB7‐dependent mitophagy
Source: EMBO J. 2017 Nov 20;37(2):235–54. doi: 10.15252/embj.201797128 (PMC5770787; doi:10.15252/embj.201797128)

Figure EV1F: RAB7 KO control blot

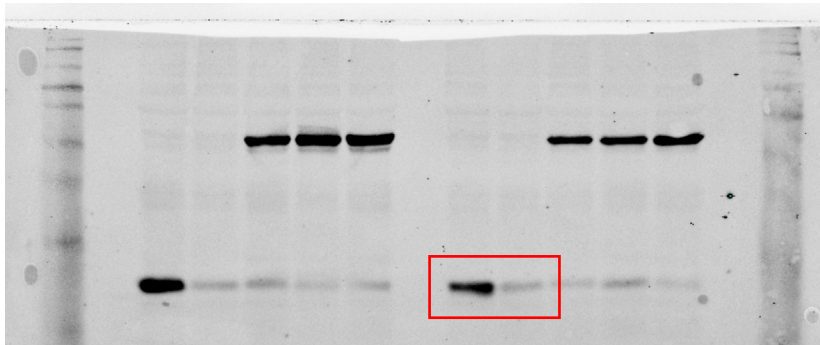

RAB7

same membrane as above, Tubulin in 680nm channel:

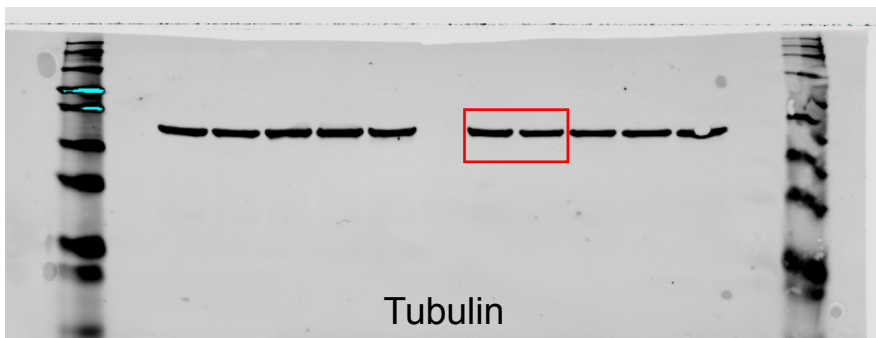

Tubulin

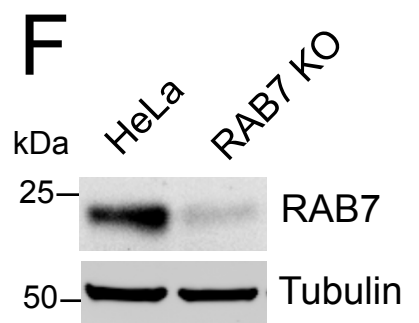

Supplement: Supplementary file 5 — Source Data for Expanded View [file EMBJ-37-235-s011.zip › Figure_EV1_blot_data.pdf]

Figure EV2A: Mito pulldown KIT

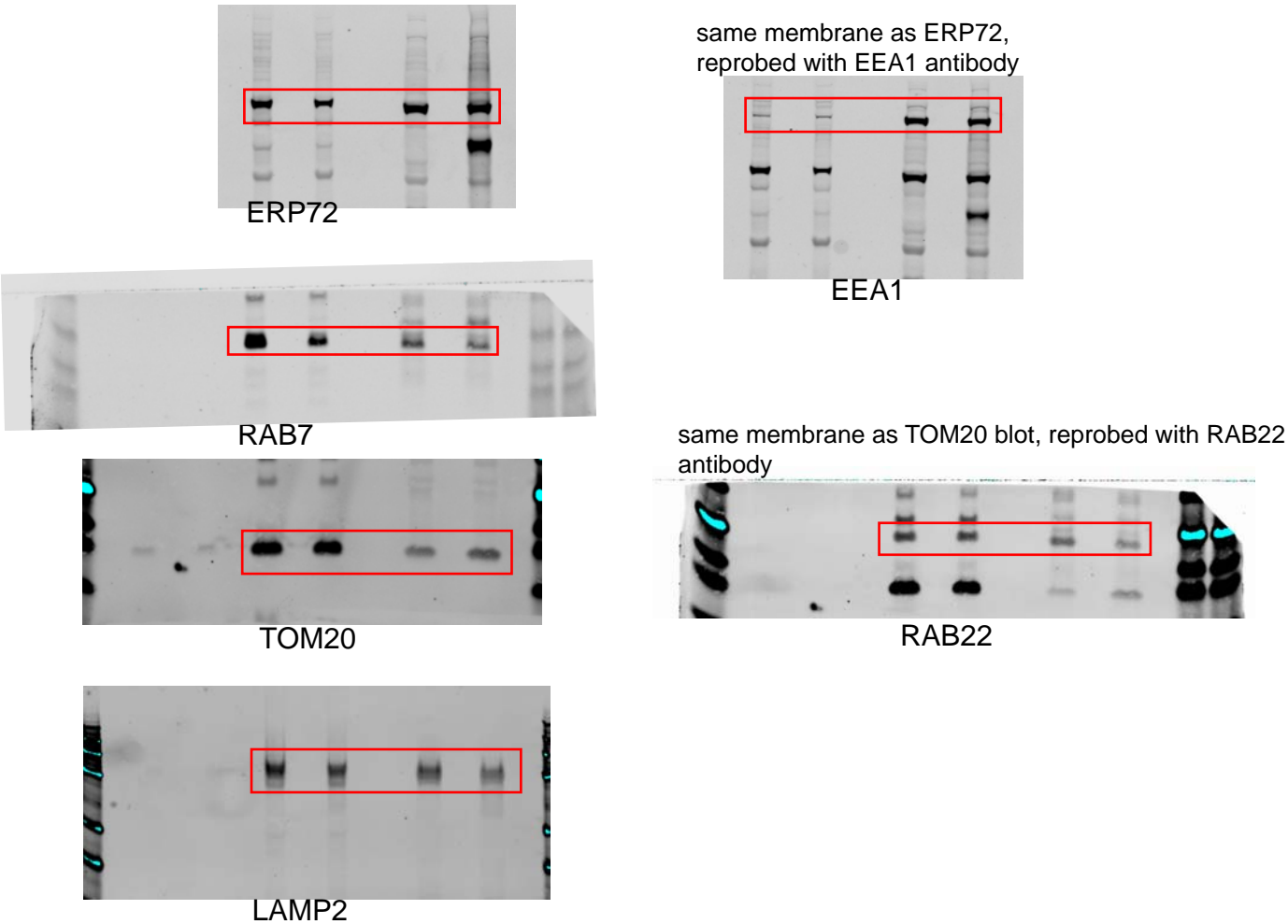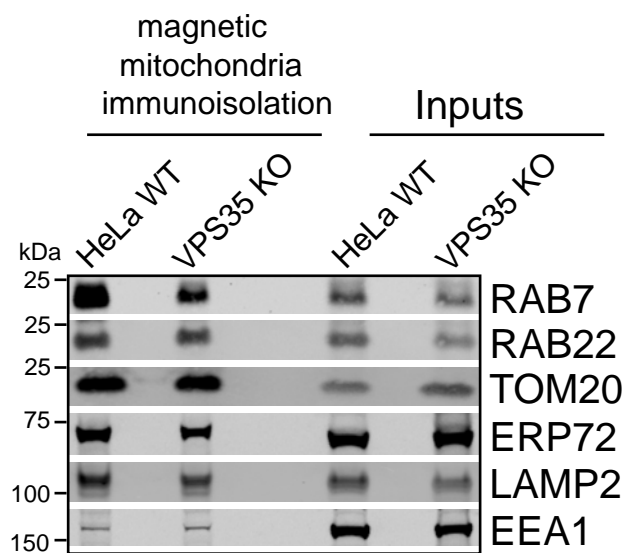

Supplement: Supplementary file 5 — Source Data for Expanded View [file EMBJ-37-235-s011.zip › Figure_EV2_blot_data.pdf]

Figure EV5C: RAB7 KO and re-expression control

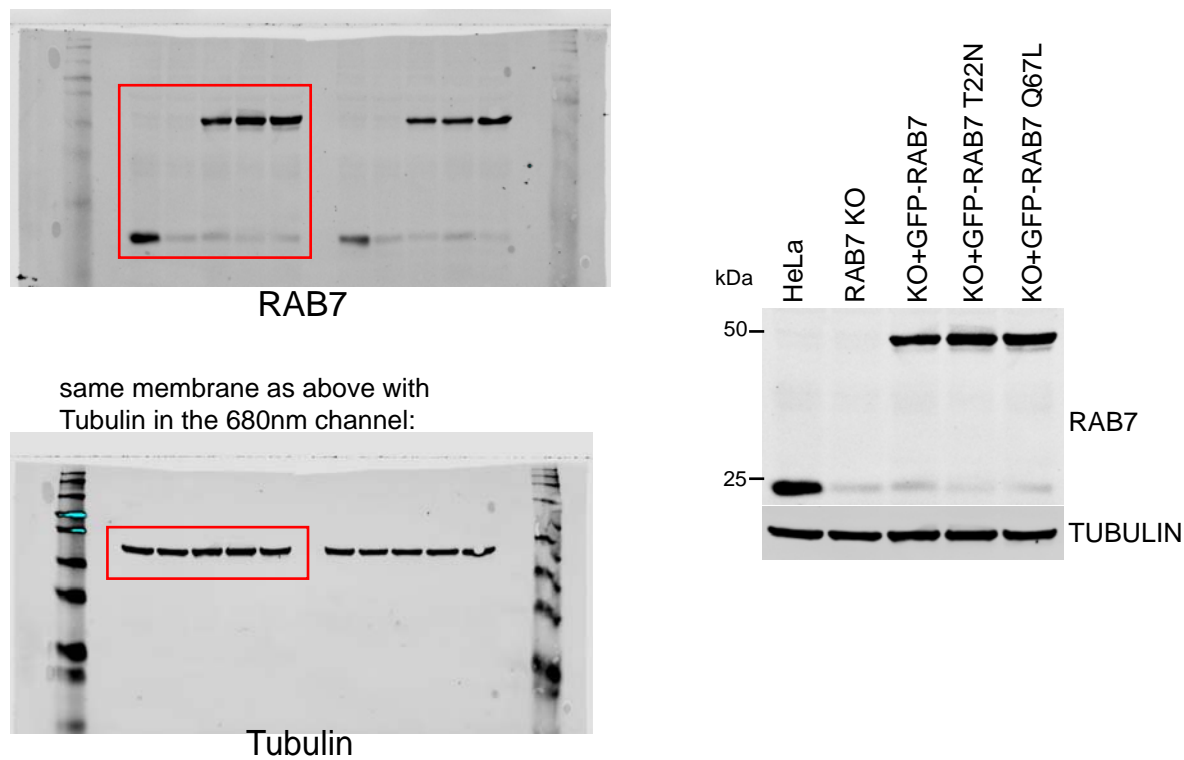

Figure EV5D: Autophagic flux

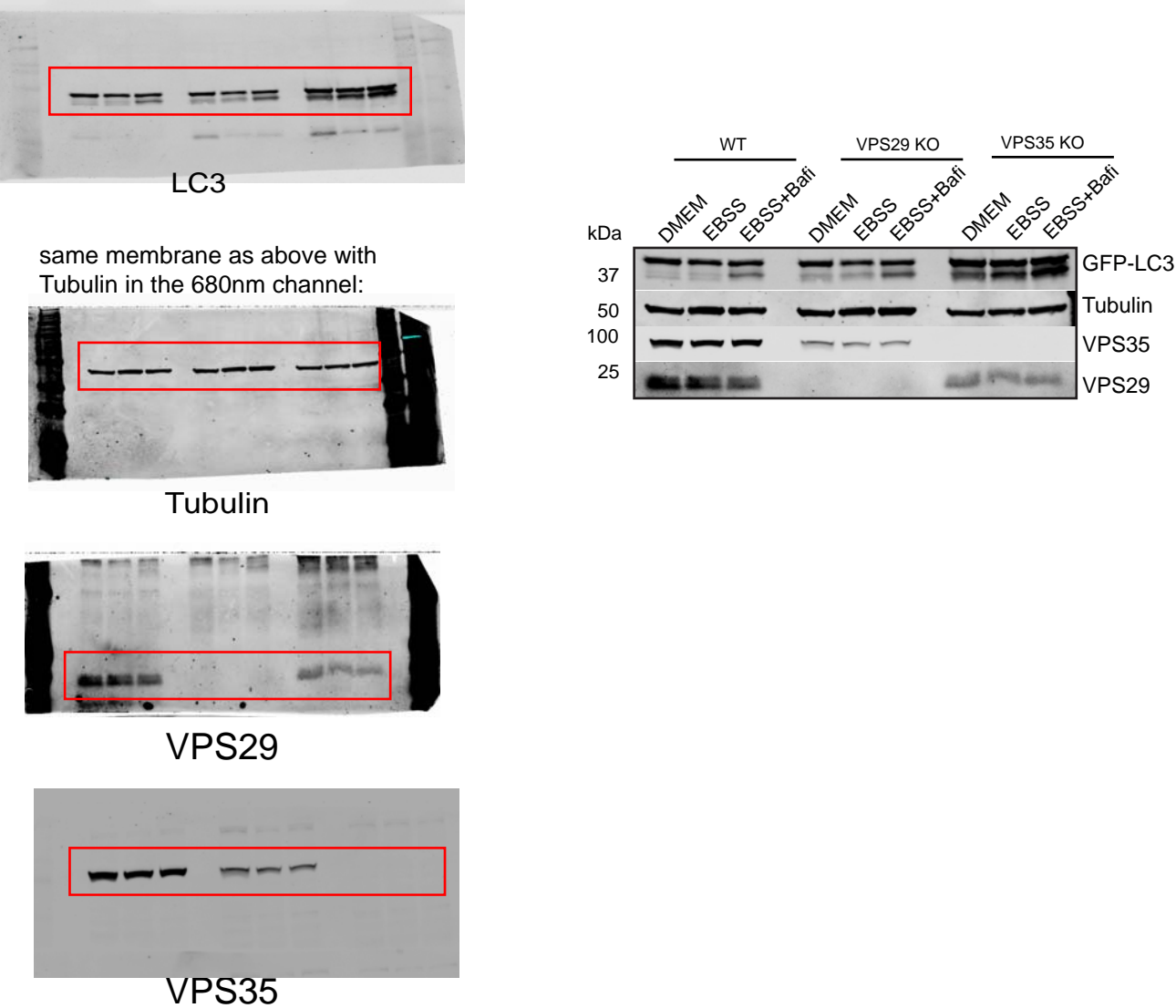

Supplement: Supplementary file 5 — Source Data for Expanded View [file EMBJ-37-235-s011.zip › Figure_EV5_blot_data.pdf]

Figure 1D: mito isolation

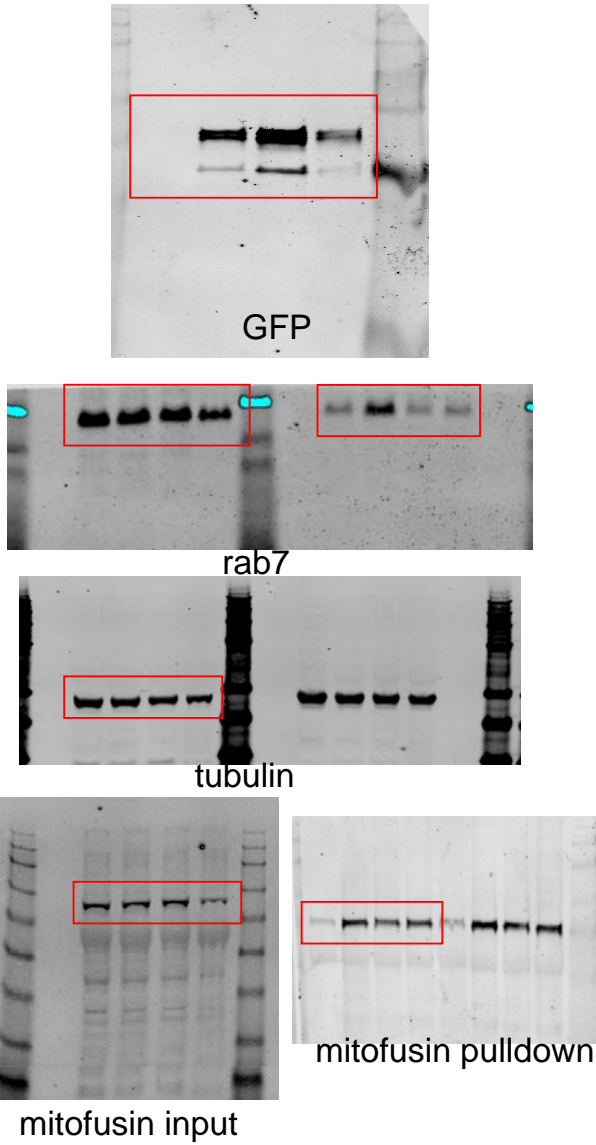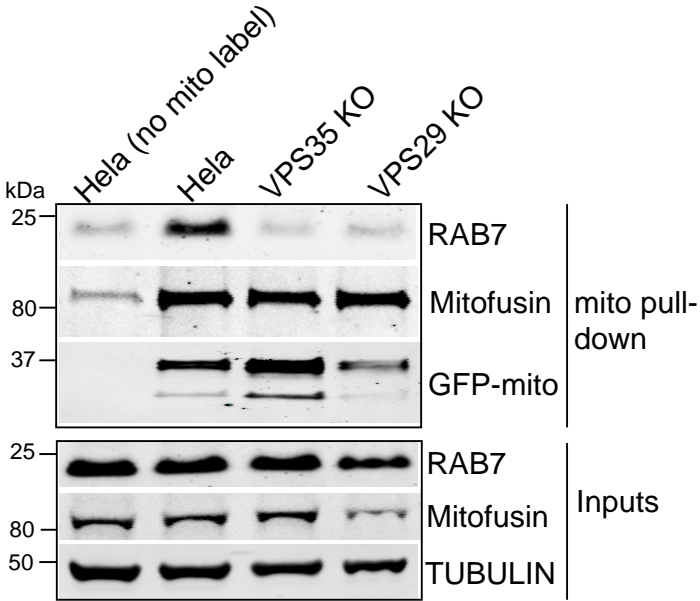

Supplement: Supplementary file 7 — Source Data for Figure 1 [file EMBJ-37-235-s005.pdf]

Figure 3B: CRISPR Screen

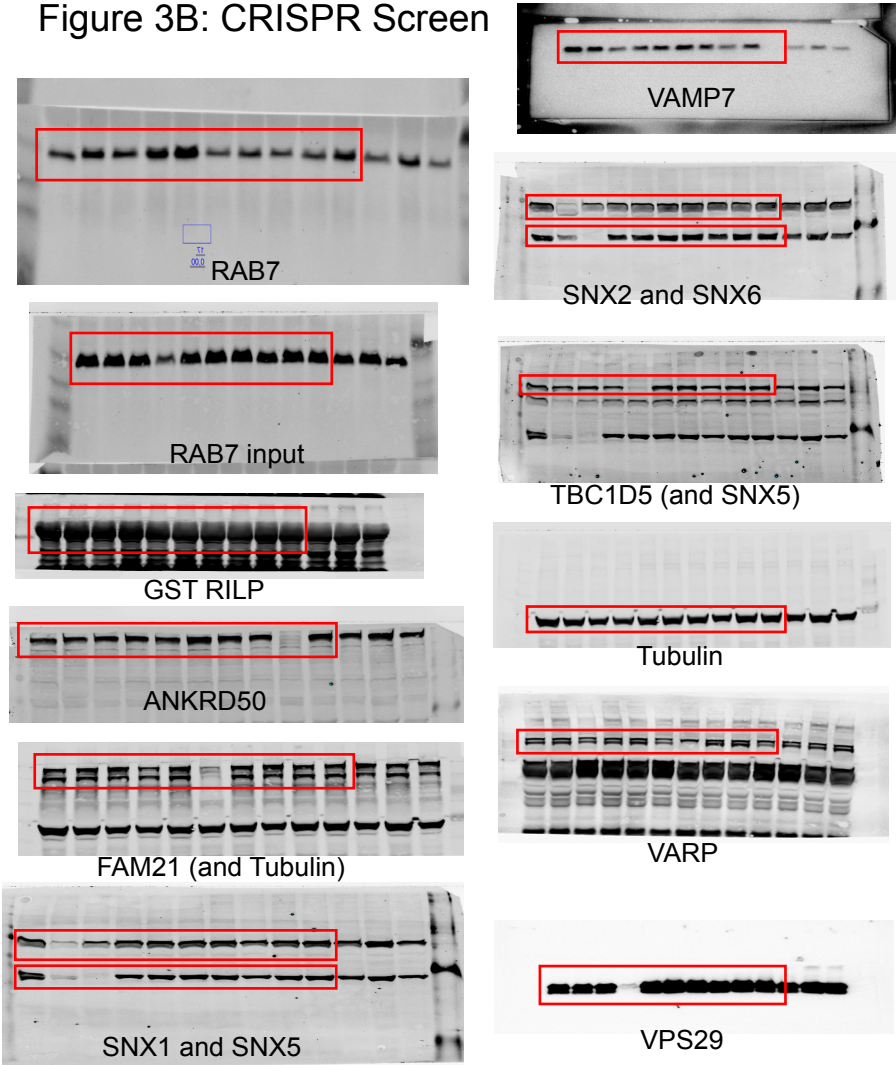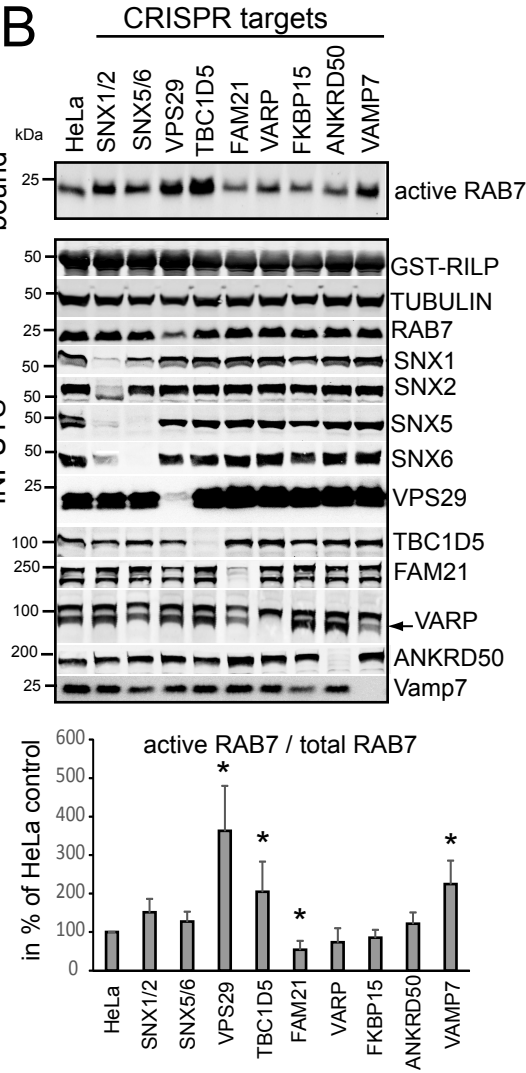

Figure 3D TBC1D5 control blot:

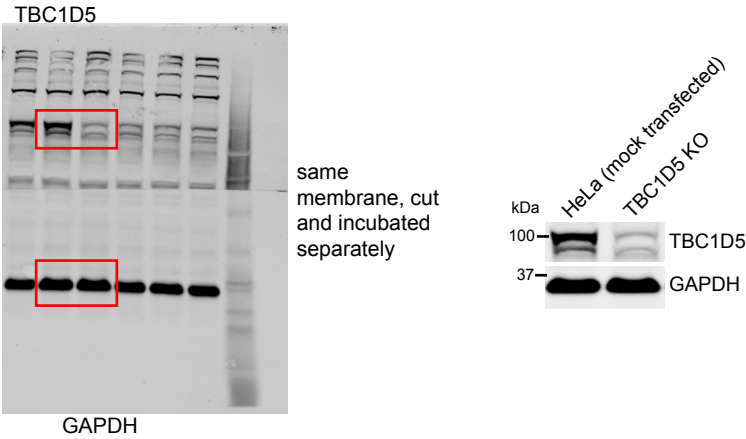

Supplement: Supplementary file 9 — Source Data for Figure 3 [file EMBJ-37-235-s007.pdf]

Figure 5B: GLUT1  
surface rescues:

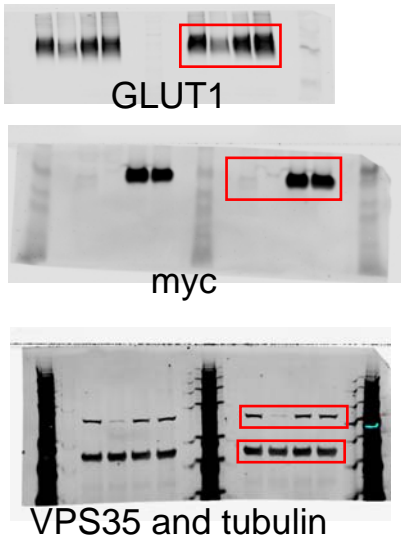

same membrane as myc blot , developed with  
VPS29 antibody and ECL:

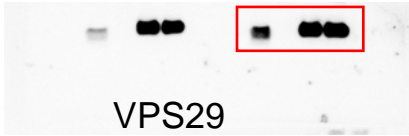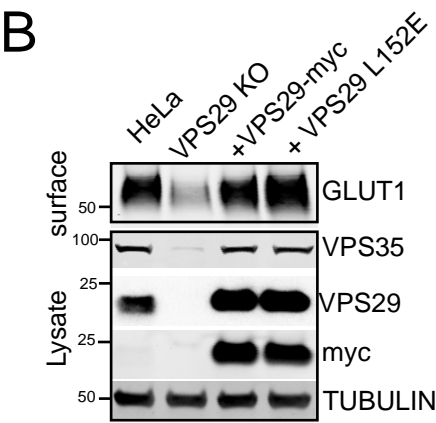

Supplement: Supplementary file 11 — Source Data for Figure 5 [file EMBJ-37-235-s009.pdf]
