# Supplementary material for: Control of RAB7 activity and localization through the retromer‐TBC1D5 complex enables RAB7‐dependent mitophagy
Source: EMBO J. 2017 Nov 20;37(2):235–54. doi: 10.15252/embj.201797128 (PMC5770787; doi:10.15252/embj.201797128)

Figure 2B: VPS35 RILP assay

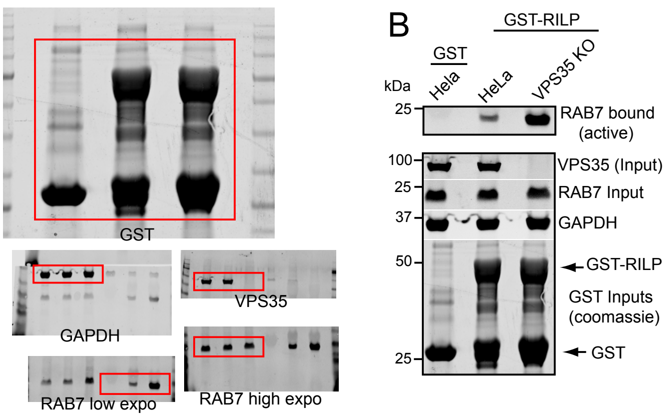

Figure 2C: VPS29 RILP assay

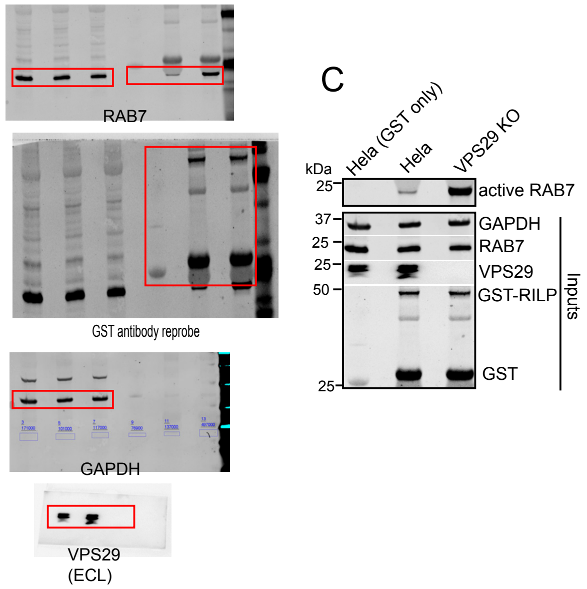

Figure 2D: VPS29 myc rescue RILP assay

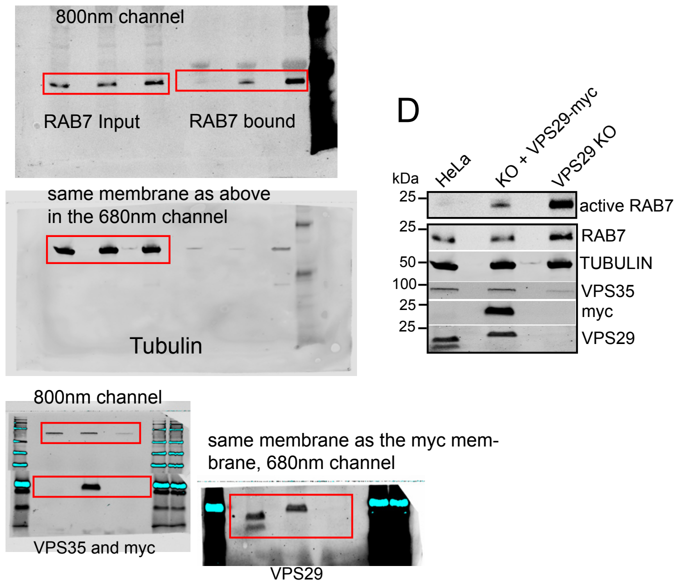

Figure 2E: RAB7 GDI2 IP

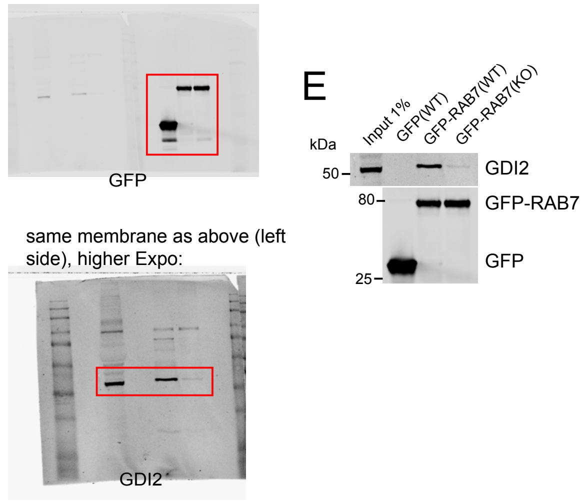

Figure 2F: GDI IP

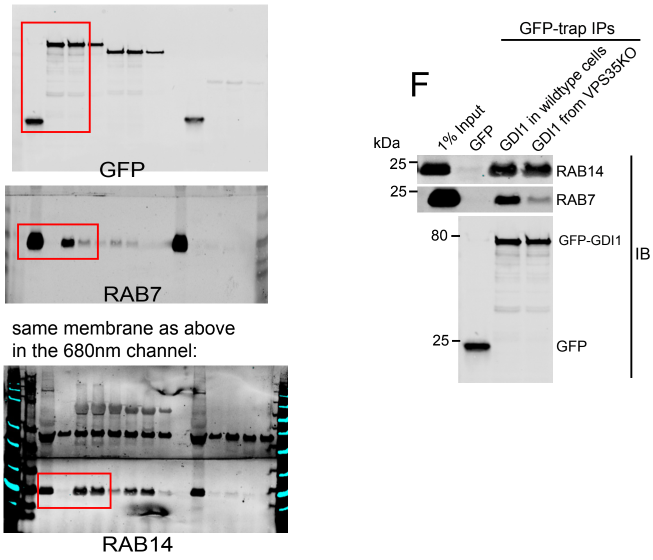

Supplement: Supplementary file 8 — Source Data for Figure 2 [file EMBJ-37-235-s006.pdf]
