# Supplementary material for: Control of RAB7 activity and localization through the retromer‐TBC1D5 complex enables RAB7‐dependent mitophagy
Source: EMBO J. 2017 Nov 20;37(2):235–54. doi: 10.15252/embj.201797128 (PMC5770787; doi:10.15252/embj.201797128)

Figure 4A: VPS29 and TBC1D5 IPs

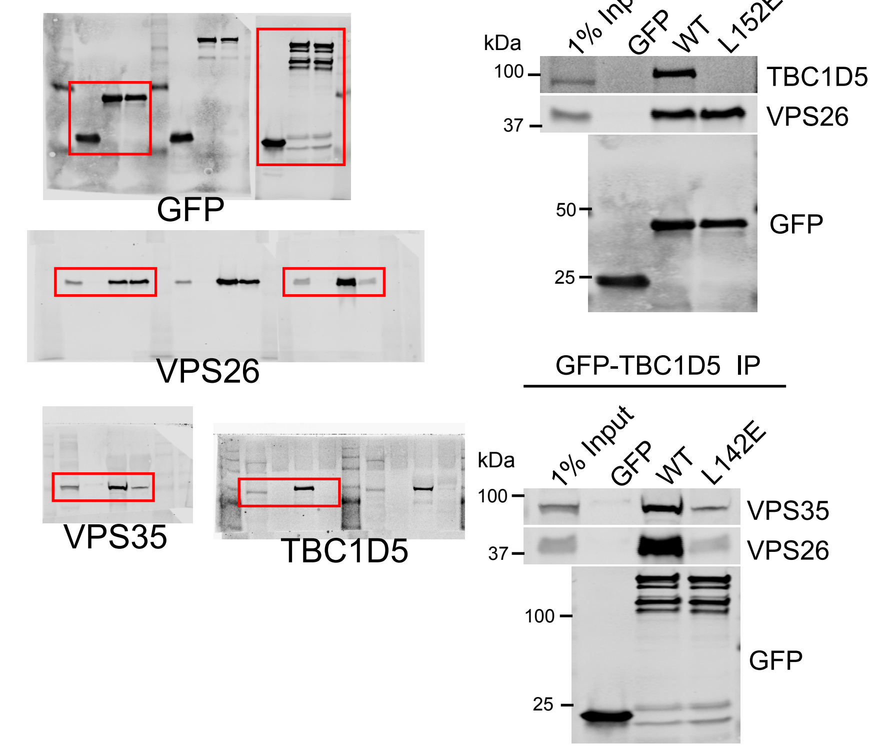

Figure 4B: VPS29 rescue RILP assay:

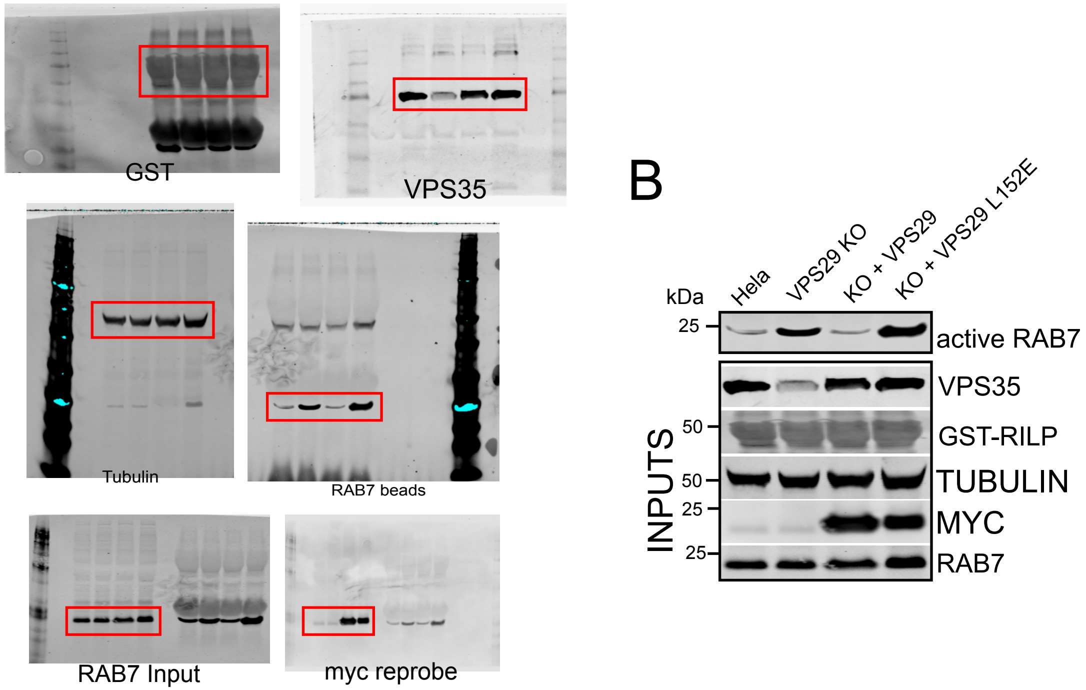

Figure 4 E: TBC1D5 KO RILP assays

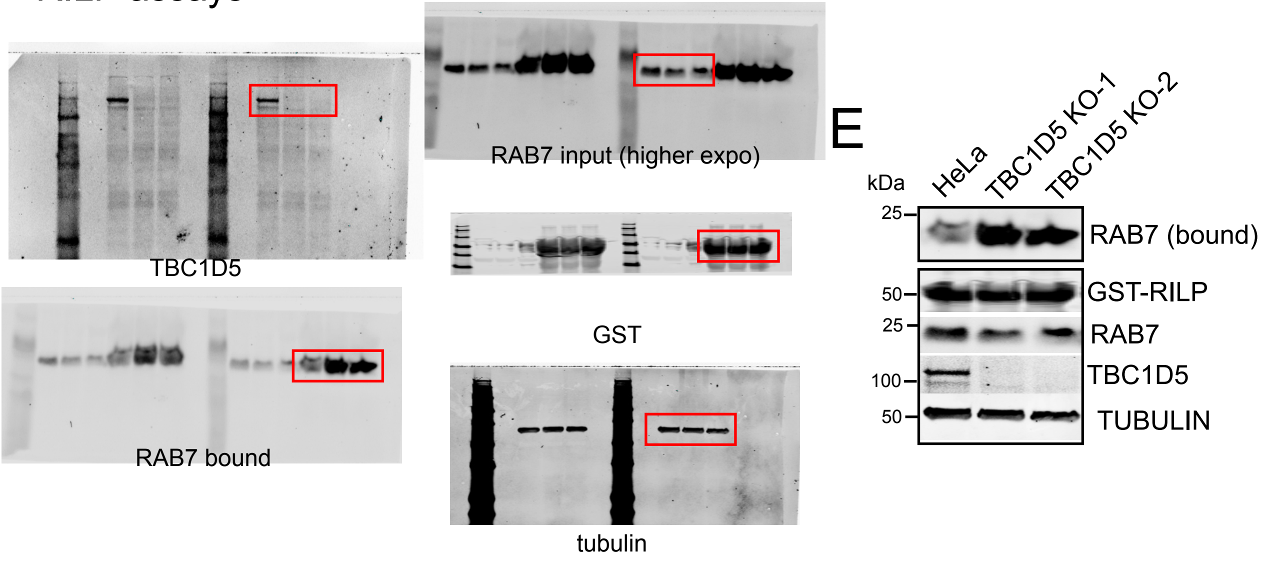

Figure 4F: TBC1D5 RILP rescues

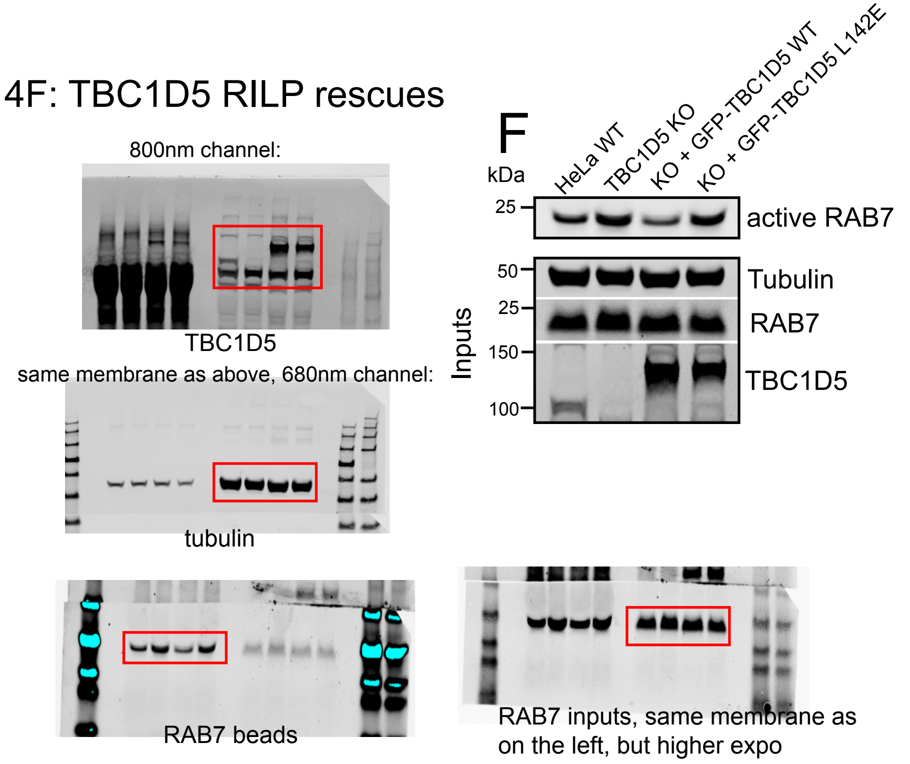

Supplement: Supplementary file 10 — Source Data for Figure 4 [file EMBJ-37-235-s008.pdf]
