# Supplementary material for: Control of RAB7 activity and localization through the retromer‐TBC1D5 complex enables RAB7‐dependent mitophagy
Source: EMBO J. 2017 Nov 20;37(2):235–54. doi: 10.15252/embj.201797128 (PMC5770787; doi:10.15252/embj.201797128)

Figure 6B: TOM20 degradation

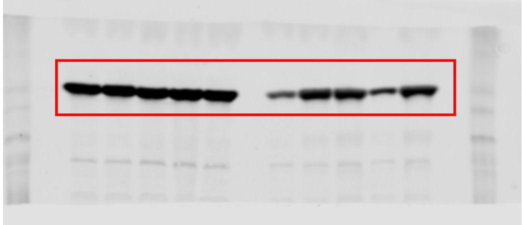

TOM20

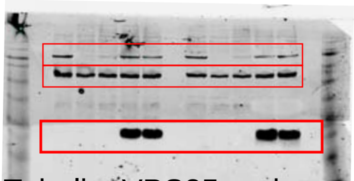

Tubulin. VPS35 and myc

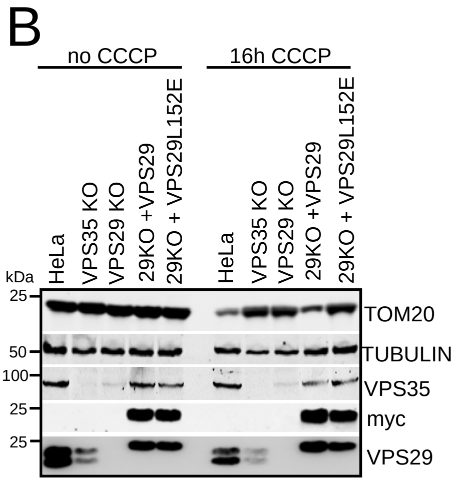

same membrane as myc blot , developed with VPS29 antibody and ECL:

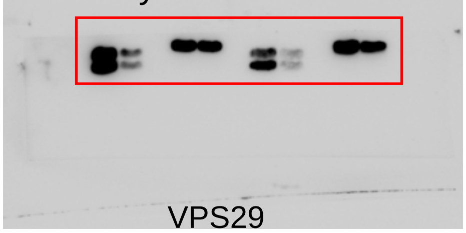

VPS29

Figure 6C: VPS35 knockdown control

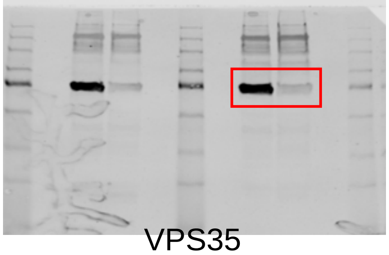

VPS35

same membrane as above in 680nm channel

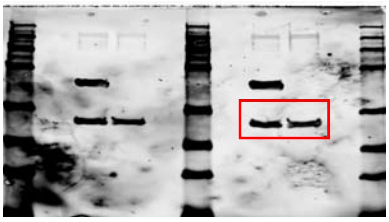

tubulin

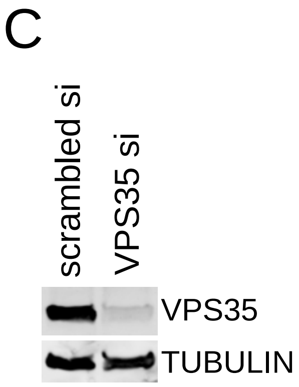

Supplement: Supplementary file 12 — Source Data for Figure 6 [file EMBJ-37-235-s010.pdf]
